# Select Venous Analytes and Fibrinogen Determination Using Two Methods in Brown Pelicans

**DOI:** 10.3390/ani14162364

**Published:** 2024-08-15

**Authors:** Amelia Gessner-Knepel, Jordan Gentry, Sharon Schmalz, Karen E. Russell, J. Jill Heatley

**Affiliations:** 1 Zoological Medicine, Small Animal Clinical Sciences, College of Veterinary Medicine & Biomedical Sciences, Texas A&M University, College Station, TX 77843, USA; aggdvm@gmail.com (A.G.-K.);; 2 Houston SPCA’s Wildlife Center of Texas, Old Katy Road, Houston, TX 77024, USA; 3 Veterinary Pathobiology, College of Veterinary Medicine & Biomedical Sciences, Texas A&M University, College Station, TX 77843, USA; krussell@cvm.tamu.edu

**Keywords:** brown pelican, fibrinogen, chemistry, bloodwork, blood gas

## Abstract

**Simple Summary:**

The brown pelican (*Pelecanus occidentalis*) is frequently impacted by both human activity and natural disasters, such as hurricanes, and is treated by rehabilitation centers. We have established normal values for juvenile brown pelicans at the end of rehabilitation to aid veterinarians and rehabilitation staff in determining if birds are healthy enough for release using analyzers readily available to these groups. We also attempted to validate a test for fibrinogen (Abaxis VSPro equine fibrinogen cartridge) in this species. Fibrinogen is a protein that can be used to measure inflammation. We found that that particular fibrinogen test is not a good way to measure fibrinogen in brown pelicans.

**Abstract:**

The brown pelican (*Pelecanus occidentalis*) is a species often affected by natural and man-made disasters such as hurricanes and oil spills, as well as general human activities; that subsequently receives medical care and rehabilitation. During rehabilitation, blood may be collected for various tests to help with diagnosis, treatment, and monitoring. Reference intervals for this species are limited, dated, and typically from small sample sizes. Seventy-one presumed healthy brown pelicans were sampled as part of their pre-release examination from rehabilitation at the Wildlife Center of Texas after a large volume stranding from December 2014 to January 2015, and various venous analytes were measured to establish updated reference intervals for brown pelicans. Fibrinogen was measured via heat precipitation and the Abaxis VSPro equine fibrinogen cartridge to determine reference intervals and in an attempt to validate the VSPro for use in avian species. Abaxis VS2 Avian/Reptile Chemistry panel, iSTAT CG4+, and iSTAT Chem8+ results, in addition to body condition score, spun PCV, cloacal temperature, and fibrinogen were measured. Proposed reference intervals for brown pelicans are presented. Fibrinogen results were not comparable between the gold standard method and the VSPro, indicating that the VSPro is not appropriate for use in brown pelicans.

## 1. Implications

The brown pelican (*Pelecanus occidentalis*) species is frequently impacted by human activities, resulting in their need for medical care and rehabilitation. However, species variation in clinical pathology analytes hinders veterinarians’ and biologists’ ability to judge the health of this species. We established blood chemistry and venous blood gas reference intervals for this species using point-of-care analyzers and fibrinogen reference intervals via heat precipitation methodology. The Abaxis VSPro Equine (Zoetis, Parsippany, NJ, USA) fibrinogen cartridge appears inappropriate for assessing fibrinogen concentrations of brown pelicans. Our findings should serve as a resource for veterinarians and rehabilitators to care for this species.

## 2. Introduction

Brown pelicans (*Pelecanus occidentalis*) are a large, protected, piscivorous waterbird species that is found along coastal areas of the Americas, extending from California to Chile, the Gulf Coast, and the Atlantic Coast from Canada to Venezuela [1]. This species was listed as federally endangered in the US in 1970 due to the pesticide dichloro-diphenyl-trichloroethane (DDT). This species remains listed as endangered in Texas despite federal delisting based on national population recovery in 2009 [2]. In the IUCN Red List, this species is now listed as least concern with an increasing population trend [1]. Along the Texas coast, this large gregarious species is often affected by natural and man-made disasters such as hurricanes, fishing, and oil spills, as well as a variety of zoonotic diseases [3,4,5]. Thus, brown pelicans are often treated by veterinarians and rehabilitators and are well suited to the investigation of clinical pathology and inflammatory indicators such as fibrinogen [6,7,8]. However, species-specific reference ranges for brown pelicans are few, dated, and limited in scope based on sample size or age [9,10,11,12]. For example, Wolf et al. [12] included 23 individuals of varying ages and sex, and Jodice et al. [11] only included trapped pelicans.

In December 2014 and January 2015, a large number of juvenile brown pelicans became stranded on the southeast coast of Texas in the Houston and Galveston areas. An excellent reproductive season, combined with a fishing tournament and stormy weather, appears to have contributed to this mass presentation. Many young pelicans were thin, lethargic, heavily parasitized, and had fishing equipment entanglement or ingestion. The pelicans were brought to the Wildlife Center of Texas for medical care and rehabilitation and were subsequently released. This relatively large population of rehabilitated, apparently healthy wild-caught birds being housed in a rehabilitation setting provided the perfect population for creating reference intervals that are most appropriate for use in rehabilitation settings.

The authors used patient-side venous blood analyzers, including the VetScan Avian/Reptile chemistry cartridge (Abaxis, Union City, CA, USA) and the iSTAT Chem8 and CG4+ cartridges (Abbott Rapid DX North America LLC, Orlando, FL, USA), to measure chemistry, electrolyte, and blood gas values. These analyzers deliver results quickly and are commonly used in wildlife, zoo practice, and rehabilitation settings [5,13]. The results were used to calculate reference intervals based on this population. The values obtained by these panels can help clinicians detect ailments, including renal disease (uric acid), hepatic disease (AST, bile acid), fluid imbalances (electrolytes and blood gas parameters), and capture myopathy. Capture myopathy has complex possible clinical pathology changes, including increased levels of AST, Creatinine, BUN, CK, lactate, potassium, and CO_2_, leading to decreased pH and acidosis [14,15,16,17].

Like most avian species, brown pelicans can hide signs of disease until death is imminent. Changes in hematologic parameters such as heterophilia indicating inflammation can occur before any overt signs of disease or merely be related to stress [5,18,19]. Thus, laboratory testing and access to species-specific reference intervals are integral to avian medicine. However, CBC determination in birds remains a laborious, non-automated process that requires training and expertise, is not well-standardized, and is often done at reference laboratories, so there is a delay between sampling and results. This delay and cost limit its utility in private practice. However, more rapid, automated, patient-side assays for fibrinogen are available for mammals. Fibrinogen testing could prove invaluable for rapid diagnosis of acute inflammation or disease if validated for use in birds. If proven valid for use in birds, patient-side fibrinogen testing may provide a lifesaving indicator of serious inflammation hours to days before CBC with less cost and expertise.

A thorough review by Davlos and Akassoglou [20] details the multitudinous roles of fibrinogen. Briefly, these include coagulation as a pro-inflammatory mediator and the sequestration of bacterial infections, among others [9,20]. Well-known diseases in which fibrinogen plays an integral role include atherosclerosis, traumatic brain injury, Alzheimer’s, colitis-associated cancer, and inflammatory bowel disease, among others. Fibrinogen increases drastically within the first 24–48 hours of inflammation and has a half-life of 3–4 days in mammals and birds [20]. This increase has been documented in numerous disease states [21,22,23]. Routine measurement of fibrinogen during diagnostic testing of birds may alert the clinician to occult inflammation [24].

Fibrinogen has been measured in a variety of avian species, including chickens, ducks, geese, penguins, ibis, flamingos, hawks, falcons, cranes, Amazon parrots, macaws, grey parrots, and owls [7,21,25,26]. Chicken fibrinogen has been shown to be similar in structure and binding sites to mammalian fibrinogen, although smaller, with the chicken fibrinogen Aα chain measuring 54,500 kDa and the human Aα chain measuring 70,900 [26,27]. In addition, the chicken and human fibrinogen molecules are catalyzed by the same coagulation factors [26]. Therefore, although the only test validated for fibrinogen determination in birds is the ‘heat precipitation’ or ‘clot recovery’ method, we suggest that fibrinogen assays made for mammals may be appropriate for use in birds [7].

The heat precipitation method, the gold standard for fibrinogen testing in humans, was utilized in this study as the gold standard method due to previous validation for use in avian species, available reference intervals, and routine use at the Texas A&M University small animal hospital clinical pathology laboratory [7]. We also used the Abaxis VSPro equine fibrinogen cartridge, an inexpensive, readily available, patient-side test that utilizes the Fibrinogen–Clauss method using a small sample size.

The goals of this study are, first, to provide updated reference intervals for healthy brown pelicans; second, to assess the agreement of two fibrinogen determination methods in brown pelicans: heat precipitation and the Fibrinogen–Clauss method via the Abaxis VSpro equine fibrinogen cartridge; third to validate the Abaxis VSPro equine fibrinogen cartridge for use in brown pelicans.

## 3. Material and Methods

### 3.1. Sample Collection and Processing

One adult and 70 juvenile brown pelicans of known gender housed at the Wildlife Center of Texas for medical care and rehabilitation for times varying from 72 h to 45 days were used for this study. The animals were maintained at outdoor ambient temperatures in 6 ft by 6 ft plywood sheet-enclosed concrete-floored pens lined with sheets and fed diets appropriate to the species. The birds were not fed that day prior to handling and were then manually restrained for pre-release physical examination, collection of cloacal swabs, and venipuncture. Physical examination parameters that were recorded include cloacal temperature, body condition score on a scale from 1 to 5 [28], wounds or other abnormalities, Wildlife Center identification band, and, when applicable, U.S. Fish and Wildlife Service band number. Sampling was performed on 31 December 2014, 6 January 2015, 21 January 2015, and 28 January 2015.

Blood samples of 1.5 mL were collected from the right jugular vein using a 3 mL syringe and a 26-ga needle. The blood was immediately transferred into Minicollect^®^ 500 μL (0.5 mL) lithium heparin pre-filled sampling tubes and 1 mL Minicollect^®^ sodium citrate microtubes (Greiner Bio-One, Monroe, LA, USA). Whole blood from the lithium heparin tubes was used on site as soon as possible to run the Abaxis VetScan Avian/Reptile chemistry panel, iSTAT Chem8, and iSTAT CG4+. The iSTAT cartridges were used based on common use in wildlife, zoo, and exotic pet veterinary medicine, even though, to our knowledge, they have only been evaluated for use in turkeys [29]. Remaining lithium heparinized blood was transported on ice to Texas A&M University’s Veterinary Teaching Hospital 2–3 h from collection sites in the greater Houston areas, where packed cell volume determination was performed immediately via centrifugation of blood in microhematocrit tubes and manual measurement using a standardized PCV determination reference card.

Upon arrival back at Texas A&M, the sodium citrate anticoagulated blood was delivered directly to the Texas A&M University Veterinary Teaching Hospital Clinical Pathology Laboratory for fibrinogen determination by heat precipitation. Within 24 h of fibrinogen testing, the sodium-citrated blood samples were retrieved from the Clinical Pathology lab and centrifuged to separate plasma from cells, and plasma was stored in a −80 °C freezer (Table 1) [30]. Based on Lewis et al. [31], fibrinogen is stable when frozen at −70 °C for five years. Sodium-citrated blood from one sampling date (21 January) was centrifuged and decanted from the cells with approximately 12 hours more delay from sampling time than all other sampling dates due to a miscommunication between authors.

### 3.2. Hemolysis Assessment

**Figure 1:**
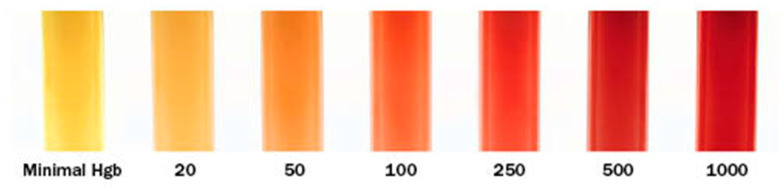
Visual hemolysis scoring chart used to estimate hemolysis in samples [32]. Hgb = Hemoglobin.

Sodium-citrated plasma was removed from the −80 °C freezer, allowed to thaw to room temperature, and assessed for degree of hemolysis prior to Abaxis VSpro fibrinogen determination via a visual assessment performed by one investigator (AGK) using the visual scale available on the Mayo Clinic Medical Laboratories website printed using a color printer [32] (Figure 1).

### 3.3. Abaxis VSpro Fibrinogen

Sodium-citrated plasma was thawed to room temperature and centrifuged using a 12 cm radius centrifuge at 3000 RPM for one hour to obtain platelet-free plasma. VSpro fibrinogen cartridges were brought to room temperature before testing. One hundred μL of plasma was mixed with diluents in the prefilled diluent microtubes provided with the cartridges. The cartridges were loaded into the VSpro, and testing was performed following the package instructions for loading the cartridge and sample into the analyzer. See Table 1 for details about testing dates. If an error message was encountered, the sample was assayed again if the sample volume was sufficient.

### 3.4. Statistical Analysis

Data analysis was performed using Analyse-it^®^ version 4.00, Method evaluation edition, (Leeds, UK), a third-party Microsoft Excel (version 2108) extension, to determine the mean, median, range, 95% confidence interval of mean, median, variance, standard deviation, coefficient of variance, Shapiro-Wilk W, and *p*-values. The Bootstrap quantile method was used to determine reference intervals, as this method does not require the assumption that data is normally distributed [33]. The Bland–Altman plot was used to assess the agreement of analytes values determined by different analysis platforms. For parametrically distributed data, ANOVA tests determined if the body condition score (BCS) had an effect on measured values. For non-parametrically distributed data, the Kruskal–Wallis test was used to determine the effect of BCS on measured values.

## 4. Results

Twelve of the birds had an injury noted on physical exam. Most injuries were minor and did not preclude release or inclusion of data for analysis. Minor injuries included bumblefoot, small lacerations or abrasions, and a fishhook in a toe pad. One bird had a severe injury to the patagium and was subsequently transported to the Texas A&M University Small Animal Hospital for medical care with the zoological medicine service. Two birds became very stressed during handling. One developed a large hematoma, and further examination was not pursued. The other died shortly after examination. Analytes obtained from these three birds were excluded from data analysis.

The mean body condition score (BCS) was 2.8 out of 5 (Median 3.0, CI 2.6–3.0) [28]. Proposed reference intervals for venous analytes from all methods used are reported in Table 2 and arranged according to the methodology. The single adult bird had values within the calculated reference intervals for all analytes. Thus, removing this individual’s data from the dataset was considered unnecessary, as it would affected the reported results. Most data was parametrically distributed as determined using a Shapiro–Wilk method. The non-parametrically distributed analytes were creatinine, aspartate aminotransferase, total calcium, uric acid, fibrinogen by heat precipitation, PCV, and total solids.

Sample size varies between each analyte due to several factors. In evaluating some samples, certain values were not read and instead reported an error message, so those values were not reported. Significant outliers in the data were excluded from the analysis. Outliers were determined using Tukey’s statistics. In addition, the Abaxis VSPro machine presented error messages with several samples. When sample volume allowed, these samples were re-run, but this did not always result in an actual reading.

The Abaxis VetScan includes albumin in the chemistry panel; however, based on Greenacre et al. [34], albumin is not reliably measured on the Abaxis VetScan, so these values were excluded. On the VetScan, blood urea nitrogen (BUN) read <0.3 on all birds. This means that this value was lower than the machine’s limit of detection. Due to this, that value is of little clinical utility due to such a low number. No *p*-value was assigned because one value was reported for all birds. This value is included for completeness.

To determine whether body condition score (BCS) affected the various values, ANOVA and Kruskal–Wallis tests were run for parametrically and non-parametrically distributed data, respectively. As BCS increased, creatinine (*p* = 0.0106) and AST (*p* = 0.0117) increased. As BCS increased, there was a decrease in sodium as measured by both analyzers (VS2 *p* = 0.0360, iSTAT *p* = 0.0057), hematocrit (*p* = 0.0122), hemoglobin (*p* = 0.0130), and anion gap (*p* = 0.0242).

**Figure A1:**
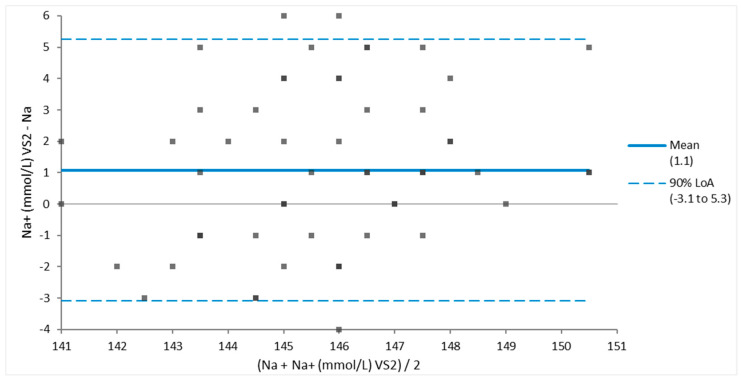
Bland-Altman Plot of good agreement for 69 sodium (Na) measurements of Brown Pelicans (*P. occidentalis*) determined via Abaxis VS2 Avian/Reptile Chemistry vs. iSTAT CG4+ cartridge. Most analyte values are within the 95% limits of agreement (LOA), the small bias is clinically acceptable, and the 95% LOA is clinically small for sodium values.

**Figure A2:**
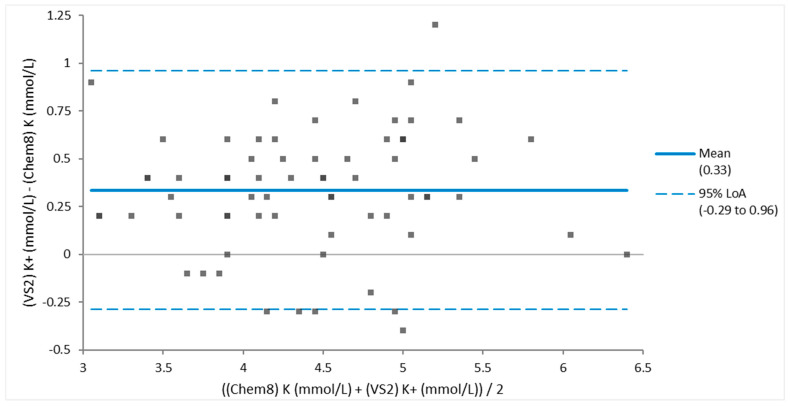
Bland-Altman Plot of the blood of 68 Brown Pelicans (*P. occidentalis*) showing good agreement of potassium obtained via Abaxis VS2 Avian/Reptile Chemistry vs. iSTAT Chem8 cartridge. Most analyte values are within the 95% limits of agreement (LoA), the small mean bias is clinically acceptable, and the 95% LoA is clinically small.

**Figure A3:**
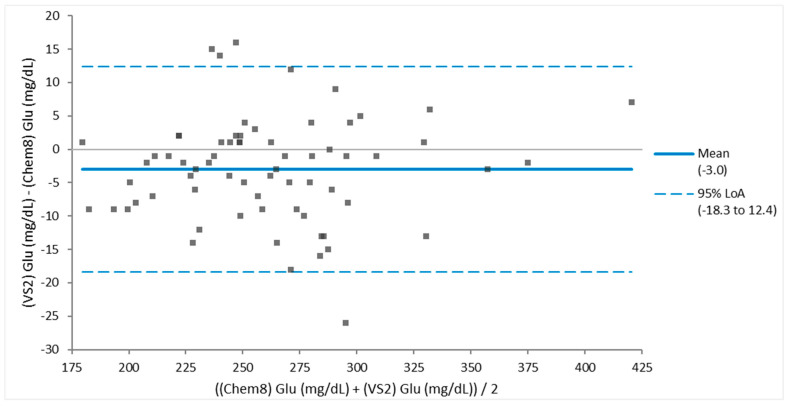
Bland-Altman Plot of blood from 68 Brown Pelicans (*P. occidentalis*) of good agreement between glucose (Glu) measurements determined via Abaxis VS2 Avian/Reptile Chemistry vs. iSTAT Chem8 cartridge. This assessment of agreement was based on the fact that all except for the one outlier lie within the 95% limits of agreement (LoA), the mean bias is clinically acceptable, and the 95% LoA is clinically small.

**Figure A4:**
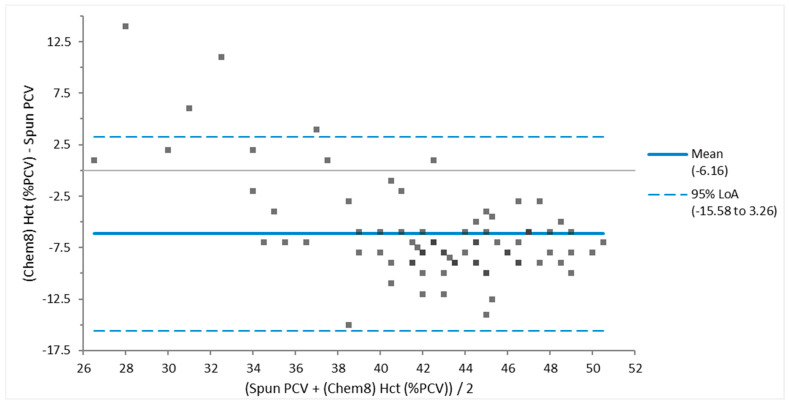
Bland-Altman Plot of Packed Cell Volume (PCV) and Hematocrit (Hct) obtained via Chem 8 iSTAT cartridge for 81 Brown Pelicans (*P. occidentalis*). Poor agreement is demonstrated based on several values outside of the 95% limits of agreement (LoA), a large clinically unacceptable mean bias of 6.16%, and a wide 95% LoA.

**Figure A5:**
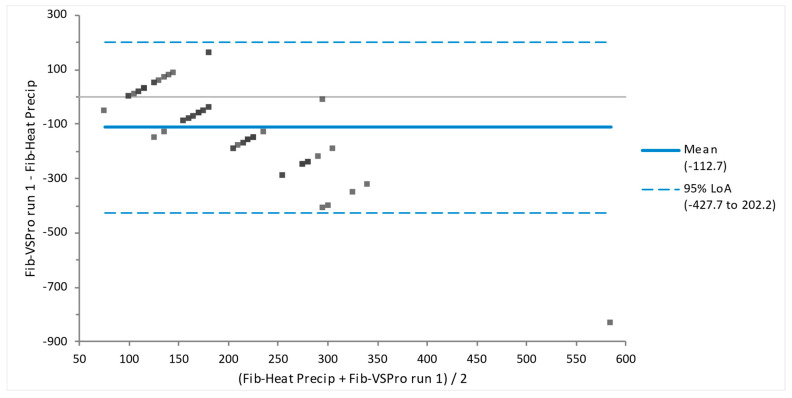
Bland-Altman Plot of minimal agreement between fibrinogen of plasma from Brown Pelicans (*P. occidentalis*, n = 62) determined based on heat precipitation (Heat Precip) versus the Abaxis VSPro Equine Fibrinogen cartridge. While most values lie within the 95% limits of agreement, the large bias was clinically unacceptable, and 95% Limit of agreement (LoA) was unacceptably wide.

Bland–Altman assessment of agreement determined good agreement in measurements of sodium (Appendix A, Figure A1) and potassium (Appendix A, Figure A2) between different analyzers. Glucose (Appendix A, Figure A3) had a fair agreement, and when one large outlier (417 mg/dL on iSTAT Chem8 and 424 mg/dL on VS2) was removed, it had a good agreement. There was poor agreement between spun PCV and hematocrit (Appendix A, Figure A4). When the two fibrinogen testing methods (heat precipitation vs. Abaxis VSPro equine Fibrinogen cartridge) were compared via Bland–Altman, and they showed no agreement (Appendix A, Figure A5). Table 3 presents a summary of Bland–Altman’s results.

Blood samples collected on different sampling dates had significantly different amounts of hemolysis, determined by ANOVA (*p* = 0.0002). However, Kruskall–Wallis testing determined that the degree of hemolysis had no statistically significant effect on fibrinogen using either methodology (*p* = 0.2249). Most tests were run on whole blood immediately or shortly after arrival at Texas A&M University. These samples should not have had the same amount of hemolysis due to more appropriate sample handling. Thus statistics were not done regarding hemolysis relating to the other values.

## 5. Discussion

According to Geffre [35] and Friedrichs et al. [36], guidelines for creating reference intervals include documenting any sources of biological variation or interference so that clinically significant factors may be controlled in the subject population. Next, establishing inclusion and exclusion criteria. Then, determining an appropriate number of individuals. The recommended minimum number of individuals is 120 to obtain 90% reference intervals in non-parametrically distributed data. However, Friedrichs [36] offers specific instructions on properly achieving quality reference intervals using fewer individuals. You can expect to achieve 90% reference intervals using at least 40 animals. Although much of our data was parametrically distributed, several values were not.

In wildlife, zoo, and exotic animal medicine, it is uncommon for a population of 120 healthy individuals to be available for reference interval determination. We were limited by our sample size of 71; however, this is a larger population size than has been used to establish reference intervals in the past for brown pelicans [10,11,12]. The listed mean values and accompanying reference intervals in Table 2 are proposed as updated reference intervals for healthy juvenile brown pelicans. The values obtained in this study are recommended for use as reference intervals for brown pelicans worldwide who are completing rehabilitation due to the large sample size and the overall apparent health of the birds. In addition, this population of pelicans was undergoing rehabilitation after stranding, which is why veterinarians frequently treat brown pelicans. Stranding may be caused by injuries due to man-made disasters such as oil spills, exposure to human objects such as boats or fishing nets and hooks or due to natural disasters such as hurricanes or prey shortages.

There was a statistically significant increase in creatinine and AST with increasing body condition score (BCS). This is likely due to increased muscle mass in these birds, which provides higher levels of protein metabolites to create creatinine and AST, as AST is derived from muscle and the liver. It was also observed that hematocrit, hemoglobin, sodium, and anion gap decreased as BCS decreased. It is suggested that these may be indicators of overall vitality, and as body condition score decreased, so did overall bodily reserves and health.

On the 21 January sampling date, significantly more hemolysis occurred than for other sampling dates. This may be because, on this sampling date, due to miscommunication between team members, separation of cells and plasma were delayed from 12 to 24 hours after sampling compared to the other sampling dates. Regardless of the cause, the hemolysis did not appear to affect the fibrinogen values, as the Kruskal–Wallis test revealed no relationship between hemolysis and fibrinogen measured by either method.

Bland–Altman analysis was used to determine whether values obtained using more than one methodology agree. When assessing Bland–Altman’s results, one must determine to what extent three criteria are met. First, whether all values are within the 95% limits of agreement; second, whether the bias is clinically acceptable; and third, whether the 95% limits of agreement range is clinically small. Values compared in this manner included sodium, potassium, glucose, packed cell volume (PCV) vs. hematocrit (Hct), and fibrinogen. Sodium, potassium, and glucose had good agreement, indicating that these different testing analyzers are comparable. The agreement criteria were not met for the fibrinogen or PCV vs. Hct. The PCV and hematocrit measurements had poor agreement. The authors recommend the use of the gold standard, manual PCV, for this value. This is because Hct is typically a calculated value, and PCV is a directly measured value. Different avian species have differently-sized nucleated red blood cells, which can impact calculations. If a methodology has not been established that can calculate the accurate red blood cell size, the Hct value is expected to be inaccurate. Fibrinogen values via the VSPro equine fibrinogen cartridge and the heat precipitation method had no agreement. It appeared that the VSPro often measured lower than the Heat Precipitation method to the extent that the bias was greater than −100.

Lipemia was encountered in only a small number of samples and was usually 1+ or 2+. One sample had 3+ lipemia. The Vetscan typically will present error messages if there is sufficient lipemia or hemolysis to impact test results. None of these error messages were encountered, so all data was included, even in cases where lipemia or hemolysis was noted in the sample.

The goal of establishing the use of the Abaxis VSPro equine fibrinogen cartridge for brown pelicans to measure fibrinogen was not achieved. The Bland–Altman analysis revealed poor agreement of these assays. The lack of agreement could be based on several factors. Firstly, chicken fibrinogen is smaller than human fibrinogen and equine fibrinogen [26]. Secondly, the Fibrinogen-Clauss method used by the Abaxis VSPro underestimates fibrinogen concentrations in humans with dysfibrinogenemia, a condition where the fibrinogen molecules are smaller than normal or abnormal in shape [37]. Also, the Fibrinogen-Clauss method uses reagents designed to determine fibrinogen in mammalian (equine) blood. Comparisons have not been made between Pelicaniformes fibrinogen molecules and chicken or mammalian. The lack of species-specific reagents and calibration could have caused falsely low readings of the Fibrinogen-Clauss method. We chose not to pursue validation of prothrombin time- (PT-) derived methods or glutaraldehyde testing based on multiple challenges inherent to these assays for use in brown pelicans. The need for species-specific reagents, the lack of fibrinogen specificity or direct quantification of fibrinogen by these assays, the sample volume needed, the time and clinical technique necessary for results, and the lack of standard curves correlating to fibrinogen concentration in avian species [37,38,39]. Instead, we assessed quantitative tests of fibrinogen, which could be directly compared, were well-validated in other species, required a reasonable sample size, and used a readily available avian-appropriate collection tube [7].

According to the instruction manual for the Abaxis VSPro equine fibrinogen cartridges, the presence of particulate matter in the blood sample can cause artifactually low readings or failure to obtain results. Particulate matter includes platelets, lipemia, or hemolysis. Based on the centrifugation of plasma samples according to VSPro manual instructions for obtaining platelet-free plasma, thrombocytes (the avian equivalent of the platelet) should not have been in the tested samples. However, a white film floating in the top layers of many plasma samples, likely to be lipemia, was apparent after centrifugation. If this layer was taken up into the pipette during sample preparation, lipids could have introduced particulates and produced a lower test result. Many of the samples that had the highest levels of hemolysis (250–500, see Figure 1) resulted in error messages, and test results were not obtained despite repeated testing. ANOVA testing of hemolysis and fibrinogen failed to reveal the effect of hemolysis on the fibrinogen value; thus, hemolysis effects upon this assay appear minimal to none.

Another possibility for the discrepancy could be sample handling. In the original manuscript where the VSPro was validated for use in equines, the blood was drawn, immediately centrifuged and decanted, and then frozen to −80 °C [30]. We used this same ultra-cold freezing temperature for storage as was used in this validation study. The plasma was thawed and used according to packaging instructions to obtain values. This method was used almost exactly, except the plasma and cells were not separated until at least twenty-four hours after the blood was collected. It may be that this extra time of plasma exposure to the cells caused changes to the fibrinogen so that it would not interact in the same way with the test reagents. In addition to the original validation study for this equipment, Fibrinogen has been proven to be stable for up to 5 years when plasma is frozen at −70 °C, so we suspect that if handling is the culprit, it was the extended time in which plasma was exposed to cells [31].

Many of our assay results are similar to previously published references for brown pelicans [11,12]. We attribute our relatively lower BUN and creatinine values to methodology, captive diet, and access to solely fresh water; our phosphorus concentrations were more similar to those of wild-caught values [12]. For calcium, our values are closer to the non-breeding captive adults than the other populations based on seasonality and geographic variations [11]. Our ranges for electrolytes more closely resemble those reported for captive freshwater pelicans. This makes sense, as most rehabilitation centers keep their animals on fresh tap water instead of salt water. Wild-caught captive juveniles appear to have electrolyte values that mimic free-living animals, aside from electrolyte parameters that seem most dependent on the available water source. The large variation in uric acid suggests a significant variation in hydration status or that some birds had recently eaten. These birds were supposed to be fasted on the day of evaluation, but the exact timing of their most recent meal was unknown. Other unknowns include whether the group was offered fresh water only or also salt water or how closely their captive diet resembled a typical wild diet in this area.

Limitations of this paper include a lack of statistical evaluation regarding the difference between male and female pelican blood values. A delay of more than 30 days occurred between the freezing of plasma samples and the Abaxis VSPro sample evaluation. Normal values for different species can vary based on housing conditions, timing of the most recent meal, hydration status, and reproductive status, among others [11]. Some of this information was not collected for our study.

We have established that, with the reported procedures, the Abaxis VSPro equine fibrinogen cartridge is not suitable for the measurement of fibrinogen in brown pelicans. We cannot assume that this test would not be suitable for fibrinogen determination in other avian species, such as Psittaciformes or raptors, or be more suitable should cells have been separated from plasma in a more timely manner.

## 6. Conclusions

In conclusion, we have presented new reference intervals that we recommend for assessing the health or disease status of juvenile brown pelicans. We have also established that the Abaxis VSPro equine fibrinogen cartridge is not suitable for use in brown pelicans.

## Figures and Tables

**Table 1:** Collection dates and sample handling data.

Collection Dates and Sample Handling Timeline			
Collection Date	Number of Birds Sampled	Time after Collection of Sodium Citrate Decanting and Freezing	Time after Collection to VSPro Fibrinogen Testing
12/31/14	24	<24 h	36 days & 53 days
1/6/15	20	<36 h	Split 39 days & 40 days
1/21/15	18	48 h	15 days & 31 days
1/28/15	9	<24 h	Split 5 days & 17 days

**Table 2:** Reference intervals for select chemistry and venous blood gas analytes of healthy juvenile brown pelicans (*P. occidentalis*) arranged by analysis method. Measured and calculated variables are included.

	Reference Intervals			
Analyte	N	Mean	95% Confidence Interval	95% Reference Interval
iSTAT Data				
Anion Gap ^1^ (mmol/L)	68	21.5	20.60–22.34	12–29
Base Excess ^1^ (mmol/L)	67	−4.4	−5.5–−3.3	−14–5
Chloride ^2^ (mmol/L)	65	108.4	107.81–109.08	103–115
Glucose ^2^ (mg/dL)	67	258	248.9–268.0	179–376
Hemoglobin ^2^ (g/dL)	68	13.4	13.05–13.68	10.5–16.0
Bicarbonate ^1^ (mmol/L)	67	21	20.1–21.9	13.4–29.4
Hematocrit ^2^ (%)	68	39.3	38.38–40.24	31–47
Ionized Calcium ^1^ (mmol/L)	60	1.26	1.226–1.287	0.99–1.52
Potassium ^2^ (mmol/L)	69	4.3	4.09–4.43	2.6–6.4
Lactate ^1^ (mmol/L)	69	8.7	7.99–9.41	3.3–16.3
PCO_2_ ^1^ (mmHg)	67	36.2	35.00–37.37	26.2–47.4
PCO_2_ ^4^ (mmHg)	65	41.3	39.94–42.66	28.7–53.6
PO_2_ ^1^ (mmHg)	69	43.3	42.0–45.0	30–61
PO_2_ ^4^ (mmHg)	67	54.4	52.29–56.52	36–76
sO_2_ ^1^ (%)	65	77.3	75.61–78.91	62–93
Sodium ^2^ (mmol/L)	68	145.5	144.90–146.02	140–150
pH ^1,5^	63	7.36	7.350–7.379	7.24–7.49
pH ^4^	65	7.33	7.310–7.342	7.19–7.49
TCO_2_ ^1^ (mmol/L)	67	22.1	21.15–22.97	14–31
TCO_2_ ^4^ (mmol/L)	68	20.3	19.60–21.02	13–28
Abaxis VS2 Chemistry Data				
Creatine Kinase (U/L)	68	1263	1150.0–1375.0	478–2511
Creatinine ^3^ (mg/dL)	66	0.34	0.321–0.364	0.2–0.5
Glucose (mg/dL)	69	258	248.5–267.9	178–374
Aspartate Aminotransferase ^3^ (U/L)	69	629.7	545.18–714.27	142–1872
Blood Urea Nitrogen *** (mg/dL)	67	<0.3	<0.3	<0.3
Total Calcium ^3^ (mmol/L)	68	10.4	10.26–10.47	9.7–11.5
Potassium (mmol/L)	70	4.6	4.41–4.76	3.2–6.4
Phosphorus (mg/dL)	68	2.8	2.54–3.04	0.3–5.2
Sodium (mmol/L)	70	146.4	145.70–147.01	141–153
Total Protein (g/dL)	69	4.6	4.45–4.67	3.8–5.8
Uric Acid ^3^ (mg/dL)	68	7.2	6.18–8.23	1.9–19.2
Fibrinogen data				
Fibrinogen-HP ^3^ (mg/dL)	70	250	218–279	100–500
Fibrinogen-VS pro (mg/dL)	59	140	133–147	70–210
Manually determined data				
PCV ^3^ (%)	66	46.4	45.23–47.59	33–54
Total Solids ^3^ (g/dL)	23	4.6	4.47–4.80	4.0–5.3
Temperature (°C)	68	104.2	103.83–104.53	100.9–107.1

^1^ This value was determined using the iSTAT CG4+. ^2^ This value was determined using the iSTAT Chem8. ^3^ This value had non-parametrically (Shapiro–Wilk) distributed variables. HP = Heat Precipitation. ^4^ This value was corrected for the patient’s body temperature. ^5^ This value was corrected for 37 °C. *** BUN measured <0.3 for all samples. Abbreviations: PCO_2_ = Partial pressure of carbon dioxide; PCV = Packed cell volume; PO_2_ = Partial pressure of oxygen; sO_2_ = Oxygen saturation; TCO_2_ = Total carbon dioxide content.

**Table 3:** Results of Bland–Altman analysis to determine agreement between two different methods of measurement for selected venous analytes in brown pelicans (*P. occidentalis*).

	Bland–Altman Results			
Parameter	n	Bias	95% Limits of Agreement	Agreement Level
Glucose VS2 vs. Chem8	67	−1.6	−30.9–27.6	Good
PCV vs. Hct	64	−6.8	−13.4–−0.1	Poor
Fibrinogen HP vs. VSPro	56	−112.0	−362.1–138.2	Poor
Sodium VS2 vs. Chem8	60	1.1	−3.9–6.1	Good
Potassium VS2 vs. Chem8	60	0.31	−0.34–0.96	Good

Abbreviations: HP = Heat Precipitation method; PCV = Packed Cell Volume; Hct = Hematocrit.

## Data Availability

None of the data were deposited in an official repository. Information on data and software used can be made available upon request.

## References

[1] BirdLife International (2018). Pelecanus occidentalis. The IUCN Red List of Threatened Species 2018: E.T22733989A132663224.

[2] DDT—A Brief History and Status. EPA Website. https://www.epa.gov/ingredients-used-pesticide-products/ddt-brief-history-and-status#:~:text=DDT%20(dichloro%2Ddiphenyl%2Dtrichloroethane,both%20military%20and%20civilian%20populations.

[3] Selman W., Hess T.J., Salyers B., Salyers C. (2012). Short-term response of Brown Pelicans (*Pelecanus occidentalis*) to oil spill rehabilitation and translocation. Southeast. Nat..

[4] Raynor E.J., Pierce A.R., Owen T.M., Leumas C.M., Rohwer F.C. (2013). Short-term demographic responses of a coastal waterbird community after two major hurricanes. Waterbirds.

[5] Kinney M.E. (2018). The effects of capture, restraint, and transport on hematologic, plasma biochemical, and blood gas values in Dalmatian pelicans (*Pelecanus crispus*). J. Avian Med. Surg..

[6] Georgieva T.M., Koinarski V.N., Urumova V.S., Marutsov P.D., Christov T.T., Nikolov J., Chaprazov T., Walshe K., Karov R.S., Georgiev I.P. (2010). Effects of *Escherichia coli* infection and *Eimeria tenella* invasion on blood concentrations of some positive acute phase proteins (haptoglobin (PIT 54), fibrinogen and ceruloplasmin) in chickens. Rev. Med. Vet..

[7] Hawkey C., Hart M.G. (1988). An analysis of the incidence of hyperfibrinogenaemia in birds with bacterial infections. Avian Pathol..

[8] Petzinger C., Larner C., Heatley J.J., Bailey C.A., MacFarlane R.D., Bauer J.E. (2014). Conversion of a-linolenic acid to long-chain omega-3 fatty acid derivatives and alterations of HDL density subfractions and plasma lipids with dietary polyunsaturated fatty acids in Monk parrots (*Myiopsitta monachus*). J. Anim. Physiol. Anim. Nutr..

[9] Zaias J., Fox W.P., Cray C., Altman N.H. (2000). Hematologic, plasma protein, and biochemical profiles of brown pelicans (*Pelecanus occidentalis*). Am. J. Vet. Res..

[10] Ferguson L.M., Norton T.M., Cray C., Oliva M., Jodice P.G. (2014). Health assessments of brown pelican (*Pelecanus occidentalis*) nestlings from colonies in South Carolina and Georgia, USA. J. Zoo Wildl. Med..

[11] Jodice P.G., Lamb J.S., Satgé Y.G., Fiorello C. (2022). Blood biochemistry and hematology of adult and chick brown pelicans in the northern Gulf of Mexico: Baseline health values and ecological relationships. Conserv. Physiol..

[12] Wolf S.H., Schreiber R.W., Kahana L., Torres J.J. (1985). Seasonal, sexual and age-related variation in the blood composition of the brown pelican (*Pelecanus occidentalis*). Comp. Biochem. Physiol. Part A Physiol..

[13] Rettenmund C.L., Heatley J.J., Russell K.E. (2014). Comparison of two analyzers to determine selected venous blood analytes of Quaker parrots (*Myiopsitta monachus*). J. Zoo Wildl. Med..

[14] Ashraf M.B., Akter M.A., Saha M., Mishra P., Hoda N., Alam M.M. (2019). Clinicopathological evaluation on capture myopathy due to chemical immobilization in spotted deer. Turk. J. Vet. Res..

[15] Breed D., Meyer L.C., Steyl J.C., Goddard A., Burroughs R., Kohn T.A. (2019). Conserving wildlife in a changing world: Understanding capture myopathy—A malignant outcome of stress during capture and translocation. Conserv. Physiol..

[16] Phillips B.E., Cannizzo S.A., Godfrey M.H., Stacy B.A., Harms C.A. (2015). Exertional myopathy in a juvenile green sea turtle (*Chelonia mydas*) entangled in a large mesh gillnet. Case Rep. Vet. Med..

[17] Spraker T., Fowler M. (1993). Zoo and wild animal medicine: Current therapy. Stress and Capture Myopathy in Artiodactylids.

[18] Cray C., Tatum L.M. (1998). Applications of protein electrophoresis in avian diagnostics. J. Avian Med. Surg..

[19] Hawkey C., Samour H.J., Henderson G.M., Hart M.G. (1985). Haematological findings in captive gentoo penguins (*Pygoscelis papua*) with bumblefoot. Avian Pathol..

[20] Davalos D., Akassoglou K. (2012). Fibrinogen as a key regulator of inflammation in disease. Seminars in Immunopathology.

[21] Harr K.E. (2002). Clinical chemistry of companion avian species: A review. Vet. Clin. Pathol..

[22] Drew M.L., Joyner K., Lobingier R. (1993). Laboratory reference intervals for a group of captive thick-billed parrots (*Rhynchopsitta pachyrhyncha*). J. Assoc. Avian Vet..

[23] Polo F.J., Peinado V.I., Viscor G., Palomeque J. (1998). Hematologic and plasma chemistry values in captive psittacine birds. Avian Dis..

[24] Godwin J.S., Jacobson E.R., Gaskin J.M. (1982). Effects of Pacheco’s parrot disease virus on hematologic and blood chemistry values of Quaker parrots (*Myopsitta monachus*). J. Zoo Anim. Med..

[25] Krajewski T., Nowak P., Cierniewski C.S. (1980). Chemical structure and properties of duck and goose fibrinogen. Biochim. Biophys. Acta (BBA) Protein Struct..

[26] Pindyck J., Mosesson M.W., Bannerjee D., Galanakis D. (1977). The structural characteristics of chicken fibrinogen. Biochim. Biophys. Acta (BBA) Protein Struct..

[27] Cardinali B., Profumo A., Aprile A., Byron O., Morris G., Harding S.E., Stafford W.F., Rocco M. (2010). Hydrodynamic and mass spectrometry analysis of nearly-intact human fibrinogen, chicken fibrinogen, and of a substantially monodisperse human fibrinogen fragment X. Arch. Biochem. Biophys..

[28] Keel Scoring Chart for Birds at North Carolina Zoo. https://nagonline.net/wp-content/uploads/2016/08/Keel-Scoring-Chart-for-Birds.jpg.

[29] Ripplinger E.N., Gruber E.J., Correa M.T., Martin M.P., Crespo R. (2023). Evaluation and establishment of reference intervals using the i-STAT1 blood chemistry analyzer in turkeys. Poult. Sci..

[30] Epstein K.L., Brainard B.M. (2012). An evaluation of the Abaxis VSPro for the measurement of equine plasma fibrinogen concentrations. Equine Vet. J..

[31] Lewis M.R., Callas P.W., Jenny N.S., Tracy R.P. (2001). Longitudinal stability of coagulation, fibrinolysis, and inflammation factors in stored plasma samples. Thromb. Haemost..

[32] Plumhoff E.A., Masoner D., Dale J.D. (2008). Preanalytic laboratory errors: Identification and prevention. Mayo Clin. Commun..

[33] Rousselet G.A., Pernet C.R., Wilcox R.R. (2021). The percentile bootstrap: A primer with step-by-step instructions in R. Adv. Methods Pract. Psychol. Sci..

[34] Greenacre C.B., Flatland B., Souza M.J., Fry M.M. (2008). Comparison of avian biochemical test results with Abaxis VetScan and Hitachi 911 analyzers. J. Avian Med. Surg..

[35] Geffre A., Friedrichs K., Harr K., Concordet D., Trumel C., Braun J.P. (2009). Reference values: A review. Vet. Clin. Pathol..

[36] Friedrichs K.R., Harr K.E., Freeman K.P., Szladovits B., Walton R.M., Barnhart K.F., Blanco-Chavez J. (2012). ASVCP reference interval guidelines: Determination of de novo reference intervals in veterinary species and other related topics. Vet. Clin. Pathol..

[37] Miesbach W., Schenk J., Alesci S., Lindhoff-Last E. (2010). Comparison of the fibrinogen Clauss assay and the fibrinogen PT derived method in patients with dysfibrinogenemia. Thromb. Res..

[38] Morrisey J.K., Paul-Murphy J., Fialkowski J.P., Hart A., Darien B.J. (2003). Estimation of prothrombin times of Hispaniolan Amazon parrots (*Amazona ventralis*) and umbrella cockatoos (*Cacatua alba*). J. Avian Med. Surg..

[39] Metzner M., Horber J., Rademacher G., Klee W. (2007). Application of the glutaraldehyde test in cattle. J. Vet. Med. Ser. A.

